# Abdominal normothermic regional perfusion in controlled DCD: toward a multidisciplinary model of organ resuscitation

**DOI:** 10.3389/frtra.2026.1930941

**Published:** 2026-08-24

**Authors:** Raphaël Giraud, Benjamin Assouline, Yvan Gasche, Franz Immer, Karim Bendjelid, Philippe Compagnon

**Affiliations:** 1 Intensive Care Division, Department of Acute Care Medicine, Geneva University Hospitals, Geneva, Switzerland; 2 Department of Anesthesiology, Pharmacology, Intensive Care and Emergency Medicine, Faculty of Medicine, University of Geneva, Geneva, Switzerland; 3 Geneva Hemodynamic Research Group, Faculty of Medicine, University of Geneva, Geneva, Switzerland; 4 Latin Program of Organ Donation, Geneva, Switzerland; 5 Intensive Care Unit, Hirslanden, Clinique des Grangettes, Chêne-Bougeries, Switzerland; 6 Swisstransplant, Bern, Switzerland; 7 Division of Transplantation, Department of Surgery, Geneva University Hospitals, Geneva, Switzerland

**Keywords:** abdominal normothermic regional perfusion, controlled donation after circulatory death, donor management, extracorporeal support, organ procurement, organ resuscitation

Dear Editors,

Controlled donation after circulatory death (cDCD) has become an essential strategy to address the persistent shortage of transplantable organs. However, organs retrieved after circulatory death inevitably undergo a period of warm ischemia prior to preservation, which exacerbates ischemia-reperfusion injury and may impair post-transplant outcomes compared with donation after brain death (1). In this context, abdominal normothermic regional perfusion (A-NRP) has emerged as one of the most promising strategies to mitigate ischemic injury in cDCD donors. By restoring oxygenated circulation to the abdominal organs after death determination, A-NRP allows metabolic recovery, physiological stabilization, and functional assessment of graft viability before procurement (2–4).

Beyond its technical implementation, A-NRP introduces a conceptual shift in donor management. Rather than representing merely a step within the procurement sequence, it creates a short but critical phase of extracorporeal organ resuscitation, during which ischemic abdominal organs are reperfused under controlled physiological conditions. After withdrawal of life-sustaining therapy and formal determination of death following circulatory arrest, in accordance with national requirements, femoral cannulation allows initiation of extracorporeal circulation, typically using a veno-arterial ECMO circuit. Mechanical occlusion of the thoracic aorta prevents thoracic and cerebral reperfusion, thereby restricting circulation to the abdominal organs (2, 3). Under these conditions, oxygenated blood is delivered at physiological temperature and pressure, allowing restoration of cellular metabolism and clearance of ischemic metabolites. Key determinants of graft recovery include adequate perfusion, sufficient oxygen delivery, correction of metabolic acidosis, and lactate clearance (3, 4).

In practical terms, A-NRP should be conducted according to a predefined institutional protocol with explicit perfusion and safety targets. Although thresholds vary between centres, commonly reported objectives include blood-based normothermic perfusion at approximately 35–37 °C, extracorporeal blood flow adapted to donor size and abdominal organ perfusion, often in the range of 3–4 L/min, and an abdominal perfusion pressure or mean arterial pressure generally maintained around 50–70 mmHg, with the important caveat that both flow and pressure should be considered together to ensure effective tissue perfusion and oxygen delivery (5). Gas exchange should be adjusted to maintain adequate oxygenation and near-physiological carbon dioxide levels, while metabolic recovery is assessed through serial pH, lactate, potassium, transaminases, urine output, and hemodynamic trends. Systemic anticoagulation should be administered according to national legislation and local protocol. Effective aortic occlusion and exclusion of cerebral reperfusion must be verified throughout A-NRP (6). Key procedural time points and safety checks should be prospectively documented and time-stamped, with independent death determination and oversight preserved throughout the process.

**Figure 1:**
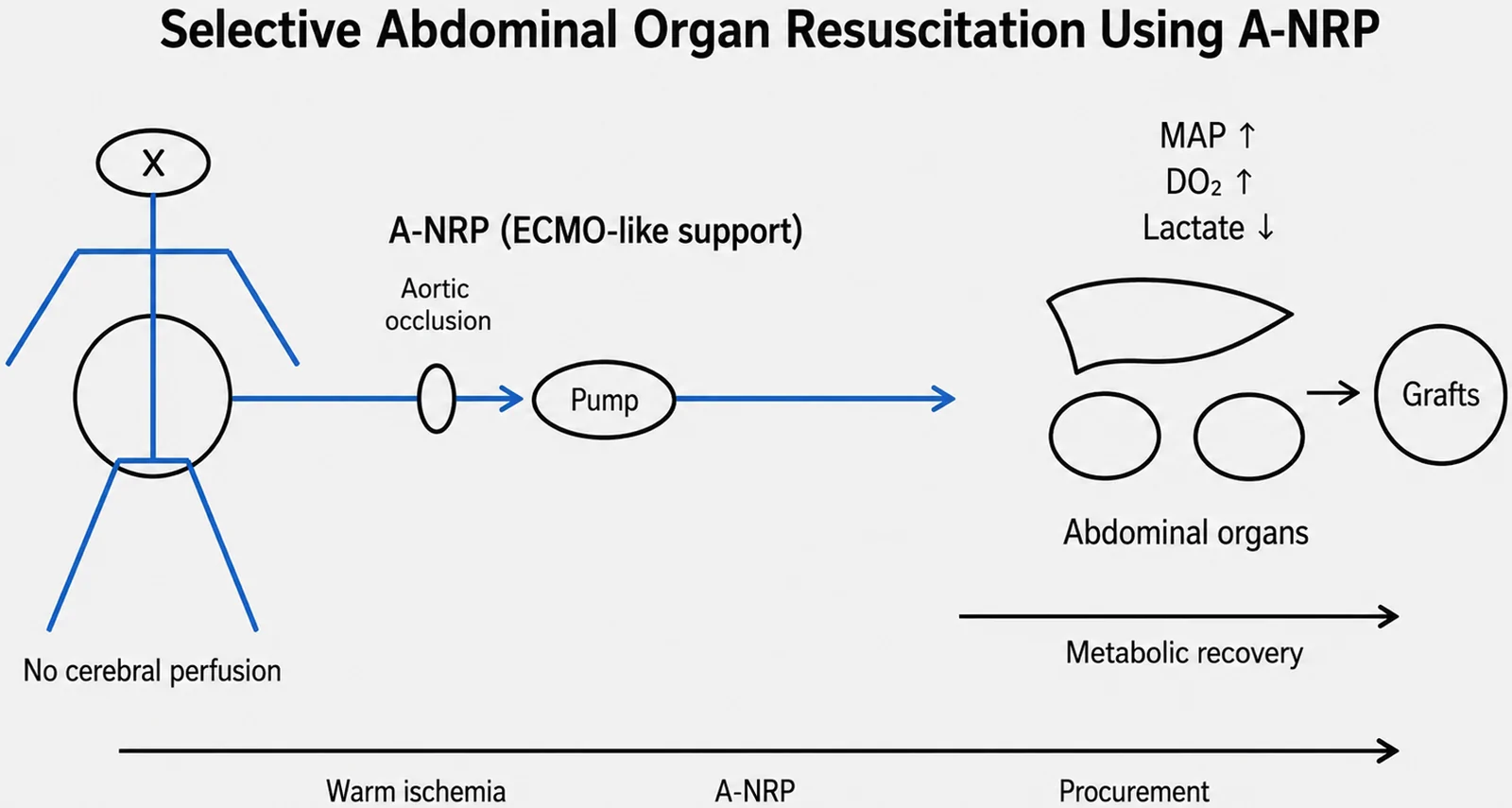
Abdominal normothermic regional perfusion as extracorporeal organ resuscitation. Following circulatory arrest in controlled donation after circulatory death, abdominal normothermic regional perfusion restores oxygenated circulation to abdominal organs through an ECMO-like extracorporeal circuit while preventing cerebral reperfusion. During this phase, perfusion pressure and oxygen delivery are restored, ischemic metabolites progressively clear, and metabolic homeostasis recovers. This short interval of controlled physiological reperfusion may transform ischemic abdominal organs into physiologically stabilized grafts prior to procurement. A-NRP, abdominal normothermic regional perfusion; ECMO, extracorporeal membrane oxygenation; MAP, mean arterial pressure; DO_2_, oxygen delivery.

In many respects, A-NRP can be understood as extracorporeal life support applied to organs rather than to patients. Much like extracorporeal membrane oxygenation is used in critical care to restore perfusion and reverse metabolic failure, A-NRP restores oxygenated circulation to ischemic organs after circulatory death. During this phase, perfusion pressure is re-established, optimized oxygen delivery is achieved, metabolic acidosis is corrected, and lactate accumulated during warm ischemia progressively clears (3, 4). From this perspective, A-NRP should not be viewed simply as a technical adjunct to organ retrieval, but as a short and decisive interval during which the physiological integrity of the graft is actively restored before procurement. This concept of A-NRP as extracorporeal organ resuscitation is summarized in Figure 1.

This interpretation is not merely semantic. It has practical implications for how A-NRP should be organized. A growing body of evidence supports its clinical benefits in cDCD transplantation. Studies from Spain and the United Kingdom have shown improved organ utilization and better transplant outcomes when NRP is used for organ recovery (4, 7, 8). In liver transplantation, A-NRP has been associated with lower rates of ischemic-type biliary lesions and improved graft survival; in Spanish cohorts, it significantly reduced biliary complications and graft loss compared with rapid cold preservation (7, 8). Kidney transplantation outcomes may also benefit from this approach, with reports showing reduced delayed graft function and satisfactory graft survival after NRP-assisted procurement (7, 9). Taken together, these findings suggest that A-NRP not only increases organ availability, but may also improve graft quality and recipient outcomes, provided that it is implemented within an appropriate legal, ethical, technical, and organizational framework.

Despite its increasing adoption, the organizational model of A-NRP remains heterogeneous across Europe. Spain has incorporated cDCD and NRP into a nationally coordinated donation framework strongly supported by intensive care teams (10). France has also developed ICU-integrated cDCD pathways in which donor management and the implementation of extracorporeal support are closely linked to intensive care practice (11). By contrast, the United Kingdom has largely developed a retrieval-team-based model in which A-NRP is frequently initiated within an operating-theatre workflow (4). Other countries have implemented regional programs adapted to their own legal and logistical constraints (7). These differences in organization are not merely logistical. They influence how A-NRP is framed within the donation pathway, either as an extension of donor management or as a component of organ procurement, and therefore shape both its ethical governance and its clinical leadership. These international experiences should not be interpreted as mutually exclusive or universally transferable models. Rather, they illustrate how A-NRP implementation is shaped by national legislation, ethical requirements, donor-management pathways, retrieval-team organization, and available institutional expertise. Any centre or country seeking to implement A-NRP must therefore adapt the procedure to its own legal and organizational framework, while preserving the core safeguards of independent death determination, prevention of cerebral reperfusion, transparent governance, and clear role separation (6).

Because A-NRP restores regional circulation after death determination, strict safeguards are required to preserve the integrity of the dead donor rule and prevent any possibility of cerebral reperfusion (12). In our view, the key ethical issue is not whether intensivists or transplant surgeons should lead the procedure *per se*, but whether a clear separation is maintained between professionals involved in pre-mortem end-of-life care and those involved in post-mortem organ resuscitation and procurement. This distinction has become more visible with the advent of A-NRP, because the transition from end-of-life care to post-mortem extracorporeal support is immediate and technically explicit.

Importantly, A-NRP has not created a new post-mortem role for intensivists. Intensive care expertise is already central to donor optimization after brain death, where post-mortem physiological support may continue for many hours, and sometimes more than 24 h, before procurement (13). Rather, A-NRP makes this post-mortem role more concentrated, more visible, and more explicitly centered on extracorporeal organ resuscitation. The ethical challenge is therefore not tied to one specialty alone, but to the need for clear separation between pre-mortem care, post-mortem organ resuscitation, procurement, and transplantation, with independent decision-making preserved throughout the process.

Because A-NRP is technically demanding and ethically sensitive, its implementation should not be reduced to the availability of an extracorporeal circuit alone. Potential failure modes include difficult vascular access, inadequate venous drainage, insufficient abdominal perfusion, incomplete aortic occlusion, inadvertent thoracic or cerebral reperfusion, circuit-related complications, contamination, and delays that may prolong functional warm ischemia. These risks justify a protocolized approach with trained operators, standardized equipment, clear fallback strategies, strict aseptic technique, real-time documentation of key procedural time points, and continuous verification that cerebral reperfusion is excluded according to local protocol (6). In this sense, safety in A-NRP depends less on a single professional group than on the reliability of the entire multidisciplinary pathway.

This same logic applies to the organizational question. Framing the issue as a binary choice between surgeons and intensivists is ultimately misleading. In some centers, the procedure is primarily performed by transplant surgeons as part of the procurement process, reflecting their expertise in vascular access and operative logistics. In other centers, particularly where NRP is initiated in the ICU, intensivists play a central role in cannulation, hemodynamic management, and metabolic monitoring. Our experience in Geneva further supports the feasibility of an ICU-integrated A-NRP pathway built on close collaboration between intensive care teams, perfusionists, and procurement surgeons. During A-NRP, physiological management relies on continuous monitoring of perfusion pressure, oxygen delivery, gas exchange, metabolic recovery, and volume status, with real-time adjustments of extracorporeal flow, vasoactive support, transfusion, fluid resuscitation, and sweep gas settings. A-NRP therefore lies at the intersection of critical care medicine, extracorporeal perfusion, and transplant surgery.

For this reason, the most robust model is not “surgeons alone” or “intensivists alone,” but a genuinely multidisciplinary framework. In such a model, intensivists contribute donor management, physiological interpretation, and ethical oversight; perfusionists ensure safe extracorporeal support and optimization of perfusion parameters; and transplant surgeons provide vascular and procurement expertise. This perspective should nevertheless be interpreted within its intended scope. The present article is a practice-oriented opinion piece rather than a systematic review, technical guideline, or comparative outcomes study. Available evidence on A-NRP remains largely based on observational cohorts, national experiences, and centre-level protocols, while legal definitions of death, consent frameworks, antemortem interventions, and organizational models differ substantially between countries. Accordingly, the model proposed here should be understood as context-dependent and conditional on robust ethical safeguards, independent death determination, adequate expertise, transparent governance, and local feasibility. Ultimately, A-NRP represents more than a technical innovation in controlled DCD. It marks the transition from passive organ preservation to active organ resuscitation before transplantation.
